# Beyond the Conventional: Successful Negotiation and Management of the Middle Mesial Canal

**DOI:** 10.7759/cureus.115564

**Published:** 2026-08-31

**Authors:** Rayaz Rasheed Moopan, Abhijith P P, Rakesh R Rajan, Sapna C Muddappa, Prabath Singh V P

**Affiliations:** 1 Department of Conservative Dentistry and Endodontics, Amrita Vishwa Vidyapeetham, Amrita School of Dentistry, Kochi, IND

**Keywords:** bio ceramic sealer, deep margin elevation, endodontics, fiber reinforced composite, magnification, mandibular molar, middle mesial canal, root canal obturation

## Abstract

Planning endodontic therapy requires a thorough understanding of the root canal system's typical morphology, anatomy, and potential abnormalities. A less common anatomical variation that requires careful investigation is the possibility of a middle mesial canal (MMC) in mandibular molars. The clinician's capacity to identify and treat such canals is greatly improved by the use of magnification. This case series emphasizes the significance of anatomical awareness in attaining predictable endodontic success by presenting clinical cases that highlight the identification and treatment of MMCs under magnification. A six-month clinical follow-up was carried out. The patients remained asymptomatic, with no reported pain or discomfort, and the treated teeth were functional. Clinical examination revealed no tenderness to percussion, swelling, or sinus tract, indicating a favorable clinical outcome.

## Introduction

The principal aims of root canal therapy are to preserve the functional integrity of the tooth, treat pulpal abnormalities, and prevent and treat periapical tissue diseases. Bacterial establishment resulting from dental caries and trauma, or iatrogenic pulp tissue exposure to oral microbiota, is the primary cause of pulpal diseases, which in turn leads to apical periodontitis. Thus, the primary objective of root canal therapy is to completely eliminate or drastically reduce the microbial load in the root canal space and minimize the risk of reinfection through effective biomechanical debridement and root canal space obturation [1].

Endodontic infections are largely initiated and sustained by oral biofilms, whose organized microbial communities and protective extracellular polymeric matrix enhance bacterial persistence and reduce susceptibility to antimicrobial treatment [2, 3].

The intricacy of root canal morphology is a significant barrier for clinicians, necessitating a detailed understanding of the anatomy of the pulp space [4]. Endodontic treatment failure can be attributed to several factors, including undetected canals, poor biomechanical preparation, insufficient instrumentation, inadequate root canal cleaning, and insufficient root canal filling [5]. When canals are left untreated, they can serve as a reservoir for ongoing intraradicular infection, a factor linked to higher rates of periapical pathology in teeth that have already undergone root canal therapy, underscoring the need for thorough identification and management of the complete canal system [6].

Among all teeth, the mandibular first molar (M1M) is the tooth most commonly treated endodontically. The middle mesial canal (MMC), taurodontism, the middle distal canal (MDC), the isthmus, and the radix paramolaris and entomolaris are among the several anatomical variants that M1M exhibits [7]. While a great deal of research has been conducted on the internal architecture of mandibular molars, middle mesial (MM) canals are not widely understood. The MM canal is a tiny, hard-to-see orifice located deep in the developmental groove or isthmus that joins the mesiolingual (ML) and mesiobuccal (MB) canals [8].

Failure to recognize and adequately manage an MMC may leave portions of the root canal system untreated. Therefore, careful examination of the pulpal floor, appropriate radiographic assessment, and the use of magnification and illumination can facilitate identification of these anatomical variations. Recent clinical reports have further highlighted the successful location and treatment of MMCs using dental operating microscopy and ultrasonic instrumentation [9]. In recent years, researchers have begun exploring artificial intelligence-driven methods for automatically detecting MMCs using cone-beam computed tomography (CBCT) imaging, with early results showing strong diagnostic accuracy and suggesting that these tools could serve as valuable aids in identifying anatomically complex canals [10].

Despite the available anatomical and imaging-based literature on MMCs, clinical reports demonstrating their identification, negotiation, and management in different clinical presentations remain relatively limited. The present case series describes three mandibular molars with confluent MMCs that were identified and managed using a dental operating microscope and adjunctive techniques. These cases emphasize the importance of anatomical awareness, enhanced visualization, and conservative canal negotiation in the clinical management of this uncommon anatomical variation.

## Case presentation

Case 1

A male patient in his early 30s sought dental care due to severe pain in the lower left posterior tooth region that had persisted for one week. The patient reported a history of severe throbbing pain exacerbated by cold beverages. The patient also reported a history of initiation of root canal treatment for the concerned tooth at a private dental clinic 4 weeks earlier. On clinical examination, a temporary restoration was present in relation to tooth 37. On radiographic examination, a radiopaque material was seen in the coronal aspect, suggestive of a restoration in relation to tooth 37. The clinical and radiographic findings were used to diagnose previously initiated therapy with symptomatic apical periodontitis in relation to tooth 37. 

Protocol for Treatment 

Informed consent was obtained from the patient before commencement of therapy. An inferior alveolar nerve block (IANB) was administered using 2% lignocaine hydrochloride (HCl) with 1:200,000 units of adrenaline (Lignox, Indoco Remedies Ltd., Mumbai, Maharashtra, India). Rubber dam isolation was performed using a HySolate dental dam (Coltene, Altstätten, Switzerland), and the temporary restoration was removed. Remaining caries excavation was performed using a BR-46 bur (Mani, Japan), and the access cavity was refined using an Endo-Z bur (Dentsply Sirona, North Carolina, United States). A DG-16 explorer (GDC Fine Crafted Dental Pvt. Ltd., India) was used to locate the canal orifices. Initially, three canal orifices were located: two mesial and one distal. Enhanced magnification using a Prima dental operating microscope (Labomed, Los Angeles, California, United States) revealed a tiny bleeding point between the MB and ML canals. A size 6 K-file (Mani, Japan) was used for canal negotiation, and patency was achieved. Following negotiation of the suspected MMC, a working-length radiograph demonstrated three distinct mesial canal paths corresponding to the MB, MMC, and ML canals. An electronic apex locator (Root ZX II; J. Morita, Japan) was used to determine the working length.

Cleaning and shaping were carried out using HyFlex EDM (Coltene, Altstätten, Switzerland) up to 25%-4 in the MB and ML canals, 20%-4% in the MMC, and 25%-6% in the distal canal. Canal irrigation was performed using 5.25% sodium hypochlorite solution (SAFE ENDO, India) and Smart Prep Liquid (SAFE ENDO, India). Irrigant activation was performed using an Ultra X ultrasonic activator (Eighteeth, Guilin, Guangxi, China). Following final irrigation, the canals were dried using sterile absorbent paper points corresponding to the final apical preparation. The canals were not excessively desiccated; successive paper points were used until the last one came out damp rather than visibly wet.

Root canal obturation was carried out using warm vertical compaction with a bioceramic sealer (Bio-C Sealer, Angelus, Brazil), followed by thermoplasticized gutta-percha (GP) backfill using the Fi-G Obturation System (Guilin Woodpecker Medical Instrument Co., Ltd., Guilin, China). Radiographic evaluation performed after obturation indicated that the MMC joined the MB canal in the middle third. Excess GP and remaining sealer were cleaned using isopropyl alcohol. The GP was sheared off 1 mm below the CEJ level and compacted using a GDC NiTi plugger (size 0.6/1 mm). One to two increments of flowable composite (Tetric N-Flow, Ivoclar Vivadent, Schaan, Liechtenstein) were placed over the access cavity. After curing the composite, dentin reinforcement was performed with fiber-reinforced composite (everX Posterior, GC Corporation, Tokyo, Japan). Core buildup was performed using packable composite (NeoSpectra ST HV, Dentsply Sirona, Konstanz, Germany) (Figure 1).

**Figure 1:**
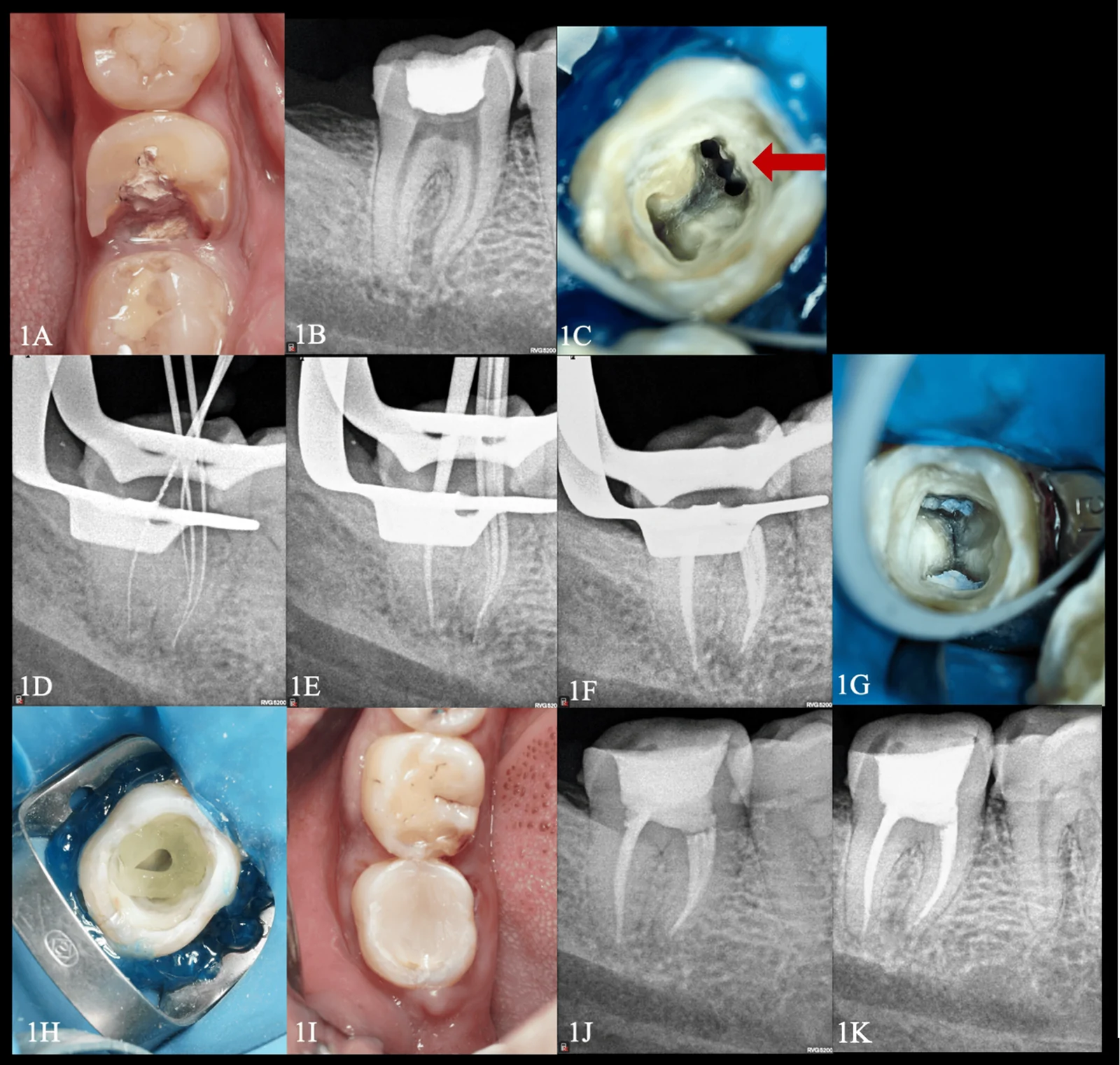
Identification, treatment, and six-month follow-up of the MMC in relation to 37 1A: Pre-treatment clinical picture. 1B: Pre-treatment intraoral periapical radiograph. 1C: Clinical view under a dental operating microscope showing the MB, MMC, and ML canal orifices; the red arrow indicates the MMC orifice. 1D: Working length determination of the MB, MMC, and ML canals. 1E: Master cone selection for the identified canals. 1F: Post-obturation intraoral periapical radiograph demonstrating the obturated mesial canals and convergence of the MMC with the MB canal in the middle third. 1G: Clinical view showing the obturated MB, MMC, and ML canals. 1H: Dentin reinforcement with fiber-reinforced composite. 1I: Post-treatment clinical picture following definitive coronal restoration. 1J: Immediate post-treatment intraoral periapical radiograph. 1K: Six-month follow-up intraoral periapical radiograph. MB: mesiobuccal; MMC: middle mesial canal; ML: mesiolingual

Case 2

A male patient in his early 20s attended the department with intense pain and food impaction in the lower left posterior tooth region, which had been present for the previous week. The patient reported a history of placement of a restoration in relation to the concerned tooth a few weeks prior to presentation. On clinical examination, a fractured restoration was seen in relation to tooth 37, with a missing distoproximal wall. On further clinical examination, the defect was found to extend subgingivally. On radiographic examination, radiopaque material was seen in the coronal aspect, suggestive of a restoration. The tooth was diagnosed with symptomatic irreversible pulpitis with symptomatic apical periodontitis in relation to tooth 37 based on clinical and radiographic examination.

Before initiation of endodontic treatment, deep margin elevation was performed using the “matrix-in-matrix” technique to raise the subgingival margin to a more coronal location using SDR composite resin (Dentsply Sirona, Konstanz, Germany). In this case, three canal orifices were initially located, following which ultrasonic troughing was performed intermittently under magnification using an ultrasonic tip (Start-X Tip #3; Dentsply Sirona, Ballaigues, Switzerland), with frequent irrigation and inspection of the pulpal floor in a mesio-apical direction away from the furcation until the MMC orifice was identified. The troughing was limited to a maximum depth of 2 mm to minimize unnecessary dentin removal and the risk of strip perforation. The subsequent treatment procedure was identical to that described in Case 1. The post-obturation radiograph demonstrated three distinct mesial canal fillings with convergence toward the apical third, supporting a confluent configuration. Post-endodontic composite restoration was performed using packable composite (NeoSpectra ST HV, Dentsply Sirona, Konstanz, Germany) (Figure 2).

**Figure 2:**
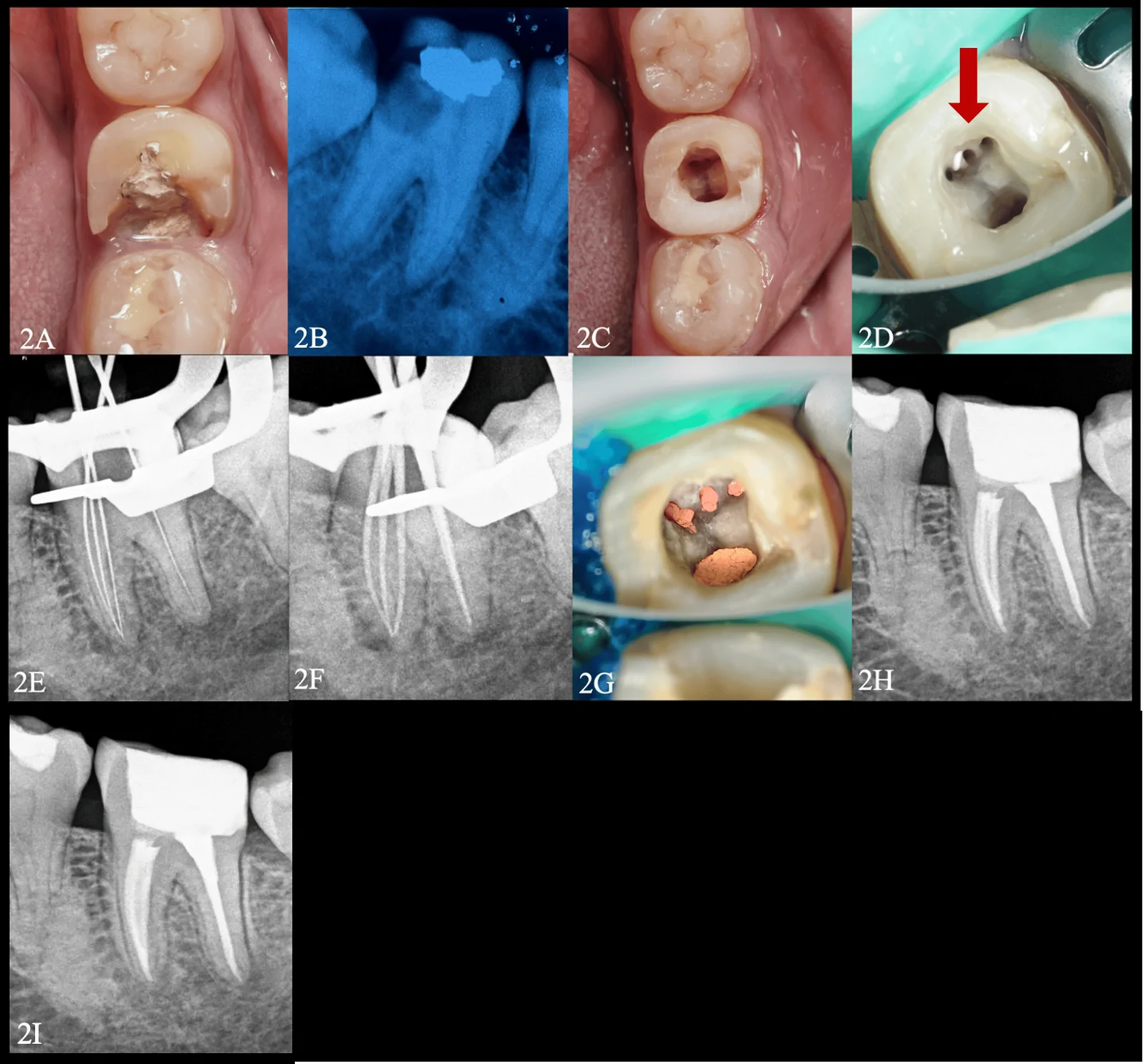
Identification, treatment, and six-month follow-up of the MMC in relation to 37 2A: Pre-treatment clinical picture. 2B: Pre-treatment intraoral periapical radiograph. 2C: Deep margin elevation and pre-endodontic composite buildup. 2D: Clinical view under a dental operating microscope showing the MB, MMC, and ML canal orifices; the red arrow indicates the MMC orifice. 2E: Working length determination of the identified canals. 2F: Master cone selection. 2G: Clinical view showing the obturated MB, MMC, and ML canals. 2H: Immediate post-treatment intraoral periapical radiograph demonstrating convergence of the three mesial canals in the apical third. 2I: Six-month follow-up intraoral periapical radiograph. MB: mesiobuccal; MMC: middle mesial canal; ML: mesiolingual

Case 3

A male in his late 40s reported with intense pain in the right lower back tooth area for a duration of one week. The patient presented with a history of initiation of root canal therapy three weeks earlier with respect to the concerned tooth. On clinical examination, a temporary restoration was seen in relation to 46. Pain on percussion and periapical palpation was noted. On radiographic examination, radiopaque material was seen in the coronal aspect, suggestive of a restoration. The diagnosis of the tooth was established as previously initiated therapy with symptomatic apical periodontitis in relation to 46 based on clinical and radiographic assessment. Temporary restoration was removed, and bleeding was noted in the developmental groove between the MB and ML canal orifices. Under dental operating microscope magnification, careful inspection of this area revealed the MMC orifice, which was subsequently negotiated and identified as an additional mesial canal. No ultrasonic troughing was used for identification of the MMC in this case. The treatment procedure implemented was identical to that described in Case 1. Sectional obturation was done in the distal canal, followed by backfill with a thermoplastic obturation system using the Calamus Dual Obturation System kit (Dentsply Sirona, United States). The post-obturation radiograph demonstrated convergence of the three mesial canal fillings at the apical level, supporting a confluent configuration. Following this, a post-endodontic composite restoration was performed (Figure 3).

**Figure 3:**
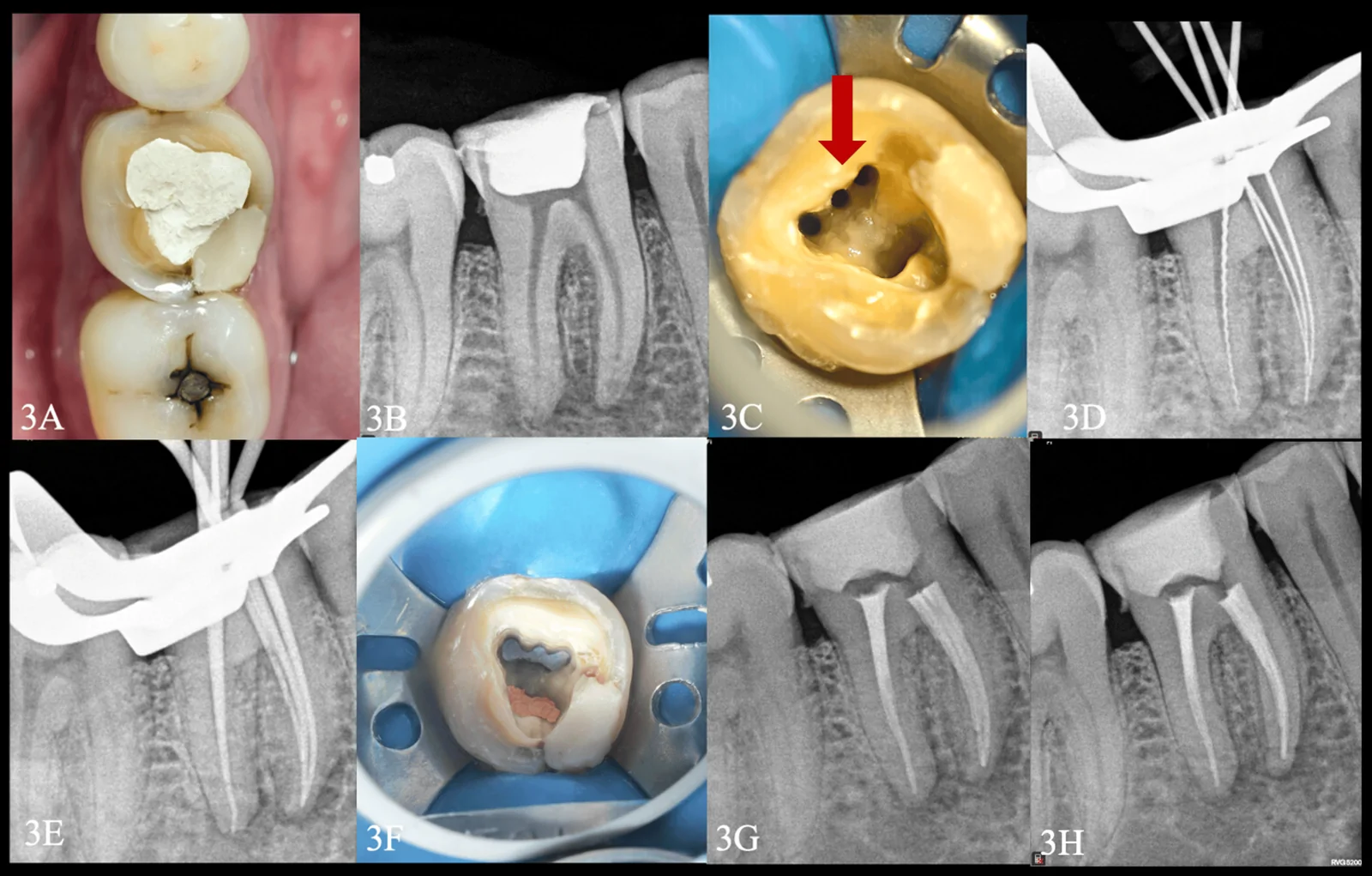
Identification, treatment, and six-month follow-up of the MMC in relation to 46 3A: Pre-treatment clinical picture. 3B: Pre-treatment intraoral periapical radiograph. 3C: Clinical view under a dental operating microscope showing the MB, MMC, and ML canal orifices; the red arrow indicates the MMC orifice. 3D: Working length determination of the identified mesial and distal canals. 3E: Master cone selection. 3F: Clinical view showing the obturated MB, MMC, ML, and distal canals. 3G: Immediate post-treatment intraoral periapical radiograph demonstrating the obturated canals and convergence of the three mesial canals at the apical level. 3H: Six-month follow-up intraoral periapical radiograph. MB: mesiobuccal; MMC: middle mesial canal; ML: mesiolingual

## Discussion

One of the biggest obstacles that clinicians encounter is the complexity of root canal morphology, which highlights the importance of a comprehensive understanding of pulp space anatomy [11]. Hess and Zurcher's early research revealed that root canal architecture is unpredictable and intricate. Multiple foramina, fins, isthmuses, apical deltas, loops, intercanal connections, and auxiliary canals are possible components of the root canal system [11].

Through a number of epidemiological and retrospective studies, Cantatore et al. demonstrated that the rate of apical periodontitis in teeth that have undergone endodontic therapy is increased when all canals are not identified and treated [12]. Missed canals may serve as a reservoir for persistent intraradicular infection and have been associated with periapical lesions in endodontically treated teeth [6].

Nosrat et al. stated that root canal morphology and form are influenced by genetic and ethnic characteristics, and these differences may be observed among different populations with varying frequencies [13]. Depending on the geographic area and methodology used, the prevalence of MMC varies considerably, with recent meta-analytic evidence demonstrating substantial variation among populations [14]. Three types of MMCs have been identified by Pomeranz et al.: fin, confluent, and independent. Based on the clinical identification of the MMCs and radiographic demonstration of convergence of the individually negotiated and obturated mesial canals, all three cases were considered to represent the confluent type [8].

The present cases further demonstrate the clinical relevance of identifying MMCs during endodontic treatment, as all three canals were successfully located and managed under dental operating microscope magnification. These cases highlight the practical importance of careful inspection of the mesial developmental groove and enhanced visualization when an additional mesial canal is suspected. Karapinar-Kazandag et al. used magnification loupes and an operating microscope to investigate the identification and negotiation of accessory mesial canals in mandibular molars. A modest enhancement in the detection of accessory mesial canals was noted, with frequencies increasing to 18% in first molars and 22% in second molars from an initial 16% in both groups. In addition, the proportion of successfully negotiated accessory mesial canals rose from 12% to 14% in first molars and from 10% to 18% in second molars [15]. Singh et al. reported the prevalence of both MMCs and MDCs in North Indian patients using a CBCT-based retrospective study, emphasizing their anatomical and clinical significance [16]. The importance of advanced imaging in locating structures that could go unnoticed was highlighted by Saber et al., who reported MMCs in 51% of the study samples [17]. Reuben et al. used spiral computed tomography and demonstrated additional canal configurations in an Indian population, further supporting the usefulness of advanced imaging in assessing complex root canal anatomy [18].

Various methods have been described in the literature for identifying MMCs, including careful anatomical inspection, multiple angulated radiographs, cone-beam computed tomography, magnification, ultrasonic troughing, micro-openers, dyes, bubble tests, transillumination, and other adjunctive techniques [19]. In the present cases, however, MMCs were identified clinically and radiographically through direct visualization under a dental operating microscope, with ultrasonic assistance used for localization in Case 2.

When instrumenting MMCs, conservative preparation is important because of their small dimensions and anatomical relationship with the surrounding root structure [20, 21]. These accessory canals should be cleaned and shaped carefully to minimize unnecessary dentin removal and the risk of procedural errors [21]. Because anatomical complexities such as isthmuses and accessory canals may limit the effectiveness of syringe irrigation alone, additional irrigant activation techniques, including passive ultrasonic and sonic activation, may be considered. Studies have demonstrated improved debris and smear layer removal with ultrasonic and sonic irrigant activation [22].

A six-month clinical follow-up was carried out for all three cases. The patients remained asymptomatic, and the treated teeth were functional, with no tenderness to percussion, swelling, or sinus tract observed on clinical examination. Six-month follow-up radiographs were also obtained for all three cases (Figures 1K, 2I, 3H), providing additional clinical and radiographic assessment of the treatment outcomes.

## Conclusions

In endodontic practice, variations in root canal anatomy continue to be a major problem that, if left unchecked, can have a direct impact on treatment results. Mandibular molars may have an MMC, which should always be anticipated. Such anatomical intricacies can be accurately identified and managed with careful clinical investigation supported by magnification. Long-term endodontic success requires knowledge of these variations and a deliberate approach to canal negotiation.
